# Levitation and controlled MHz rotation of a nanofabricated rod by a high-NA metalens

**DOI:** 10.1038/s41378-025-00886-7

**Published:** 2025-04-21

**Authors:** Hailong Pi, Chuang Sun, Kian Shen Kiang, Tiberius Georgescu, Bruce Jun-Yu Ou, Hendrik Ulbricht, Jize Yan

**Affiliations:** 1 School of Electronics and Computer Science, University of Southampton, Southampton, SO17 1BJ UK; 2 School of Physics and Astronomy, University of Southampton, Southampton, SO17 1BJ UK

**Keywords:** Nanophotonics and plasmonics, Nanophotonics and plasmonics

## Abstract

An optically levitated nanoparticle in a vacuum provides an ideal platform for ultra-precision measurements and fundamental physics studies because of the exceptionally high-quality factor and rich motion modes, which can be engineered by manipulating the optical field and the geometry of the nanoparticle. Nanofabrication technology with the ability to create arbitrary nanostructure arrays offers a precise way of engineering the optical field and the geometry of the nanoparticle. Here, for the first time, we optically levitate and rotate a nanofabricated nanorod via a nanofabricated a-Si metalens which strongly focuses a 1550 nm laser beam with a numerical aperture of 0.953. By manipulating the laser beam’s polarization, the levitated nanorod’s translation frequencies can be tuned, and the spin rotation mode can be switched on and off. Then, we showed the control of rotational frequency by changing the laser beam’s intensity and polarization as well as the air pressure. Finally, a MHz spin rotation frequency of the nanorod is achieved in the experiment. This is the first demonstration of controlled optical spin in a metalens-based compact optical levitation system. Our research holds promise for realizing scalable on-chip integrated optical levitation systems.

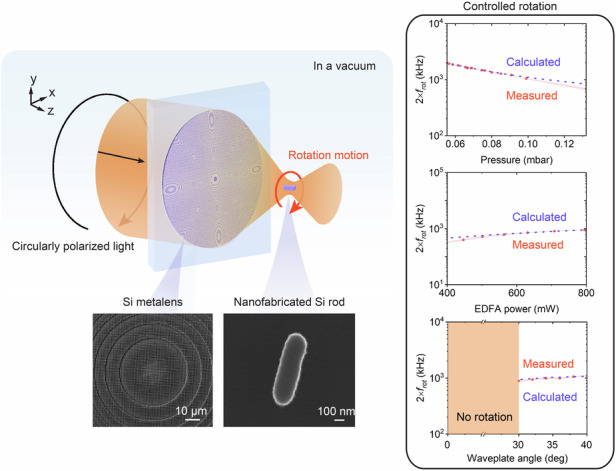

## Introduction

Optomechanics is related to utilizing light to control mechanical objects. Levitated optomechanics has become an exciting research field with the advantages of low dissipation and minimal thermal contact with the environment^1–3^. In high vacuum conditions, optical levitation systems exhibit extremely low damping loss, enabling a high-quality factor^4,5^. Levitated nanoparticles possess multiple motion modes, such as centre-of-mass (COM) motions, libration, rotation, and precession^1–3,6–8^. These advantages enable optical levitation systems to be ideal platforms for studying thermal dynamics^7^ and sensing forces^9^, acceleration^10^, and torque^11^. Recently, the advances in cooling an optically trapped nanoparticle into its quantum ground state of motion^12–15^ in a vacuum show great potential for studying macroscopic quantum mechanics^16,17^.

Arising from the light-matter interaction, the dynamics of an optically levitated nanoparticle is determined by the trapping light field and the nanoparticle’s geometry property. Manipulating the light field and nanoparticle geometry would enable the levitated system for various sensing and physics exploration scenarios. In the aspect of manipulating light field, a tunable focal length can be used to precisely control the particle along the optical axis, which could facilitate the detection of short-range interactions^18^. Multi-point focusing of light provides a research platform for multi-particle coupling^19^, cooling^20^ and on-demand assembly^21^, setting an important stage for achieving macroscopic many-body physics. Structured light enables novel control of particle’s rotational dynamics through orbital angular momentum transfer^22^ and facilitates stable trapping using a doughnut-shaped beam^23^. In the aspect of engineering nanoparticle geometry, non-spherical nanoparticles can be used to achieve fast rotation for torque measurements^6,7^. Unique structures such as prisms make it possible to search for high-frequency gravitational waves^24^ and explore polarization-based inverse optical torque^25^.

Nanofabrication technologies capable of producing nanostructure arrays offer a precise method for engineering light fields and nanoparticle geometry for optical levitation. In terms of manipulating the light field, the nanofabricated metalens would be powerful in controlling the light beam’s phase^26^, amplitude^27^, and polarization^28^. In recent years, optical trapping in liquid based on compact metalens has been reported^29–32^. Optical levitation in a vacuum faces greater challenges than in liquid, requiring a larger numerical aperture (NA) to provide a deep trapping potential well. Following a pioneering work of optically levitating one nanoparticle via a metalens with an NA of 0.88^33^, our group reported on-chip optical traps for levitating two nanoparticles via a dual-foci metalens with an NA of 0.9^34^. In terms of engineering nanoparticles, nanofabrication provides a precise way to achieve good uniformity and well-controlled size of particles. Nanofabricated silicon nanorods with controlled length and diameter have been reported^35^. Such nanorods have been optically levitated using two conventional lenses and showed controlled rotations^36^. Given these advancements driven by nanofabrication technology, a key trend in optical levitation in a vacuum is the combination of nanofabricated metalenses and nanofabricated particles. This combination can fully exploit the advantages of both metalenses and nanofabricated particles, enabling the development of highly controllable, scalable, and on-chip integrated optical levitation systems.

**Fig. 1:**
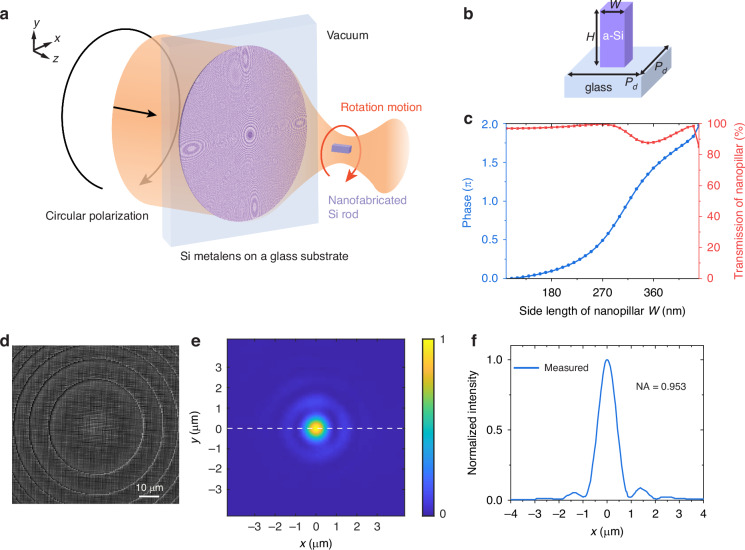
Metalens-based optical levitation system and the metalens’ performance. **a** Schematic of the optical levitation in vacuum combining the nanofabricated metalens and a nanofabricated nanorod. This figure shows that when circular polarization is applied, the particle exhibits rotational motion. In the experiments, the polarization state is also adjusted to linear polarization to study the particle’s translational motions. **b** The periodical element of the metalens: an amorphous silicon (a-Si) nanopillar on a glass substrate. *W* is the side length of the nanopillars. The nanopillar height *H* is 800 nm. Centre-to-centre distance between neighbouring nanopillars *P*_*d*_ is 600 nm. **c** Simulated propagation phase and transmission as a function of the nanopillar’s side length *W* (110–440 nm). **d** Top-view SEM image of the fabricated metalens. **e** Measured intensity distribution in the focal plane. **f** The intensity profile along the *x*-axis passes through the focus

Here, we demonstrate a fully nanofabricated optical levitation system where the trapping laser beam is strongly focused by a nanofabricated metalens with an NA of 0.953 and the trapped nanorod is nanofabricated (Fig. 1a). The nanofabrication process of the metalens and the silicon nanorods is illustrated at first. Then, a metalens-based optical levitation system is built to study the levitated dynamics of the nanofabricated nanorods. We show the manipulation of the nanorod’s translational and rotational mechanical eigenfrequencies. The nanoparticle’s rotation is the first reported rotation in a vacuum by a metalens-based optical levitation system.

## Results

### Characterization of metalens

In order to achieve the desired focusing effect, the metalens needs to impart a focusing hyperbolic phase distribution to the wavefront, as described by the following equation^37^

1 $$\varphi (R,\lambda )=-2\pi \left(\sqrt{{R}^{2}+{f}^{2}}-f\right)\frac{1}{\lambda }$$

where *λ* = 1550 nm is the wavelength, *R* represents the radial distance from the lens centre, and *f* = 200 µm is the designed focal length. A dense pattern of square amorphous silicon (a-Si) nanopillars on a glass substrate is adopted to encode the phase profile on the incoming plane wave. Each a-Si nanopillar (Fig. 1b) acts as a miniature antenna, giving a phase shift to the transmitted light. By varying the side length *W* of the pillars from 110 nm to 440 nm, we can obtain a 2*π* phase coverage while maintaining high transmission larger than 85.3% (Fig. 1c). The transmission of the nanopillar is defined as the ratio of the light power passing through a nanopillar to the initial power. The polarization used for the simulation is the linear polarization along the *x*-axis. The responses of the transmission and phase change are the same as the results in Fig. 1c when using linear polarization along the diagonal of the nanopillar’s square cross-section or circular polarization, as shown in supplementary material section S1. The nanopillars maintain a uniform height *H* of 800 nm, with a centre-to-centre distance *P*_*d*_ of 600 nm between neighbouring nanopillars. The designed and fabricated metalens has a diameter of 1.2 mm. See “Materials and methods” for the fabrication process.

In order to determine the numerical aperture (NA) of the fabricated metalens, the laser intensity distribution at the focal spot is measured. In the measurement, a 1550 nm laser with a beam waist much larger than the diameter of the metalens is used to ensure a plane wave input with a constant amplitude. The image of the focused laser beam after the laser passes through the metalens is captured by using an objective lens and a camera. Figure 1e shows the measured intensity distribution of the focus in the *xy* plane, revealing a clear and well-defined focal spot. Figure 1f presents a cut across the *x*-axis passing through the focus (dotted line in Fig. 1e), showing an Airy profile. The Airy radius (*r*_*A*_), defined as the radius from the central peak of the Airy pattern to its first minimum, is 992 nm. So the corresponding NA is 0.953, calculated based on the relationship *r*_*A*_ = 0.61*λ*/NA. The obtained NA agrees well with the designed NA (0.95). The error in measuring NA is about 2.1%, mainly from the error in estimating the Airy disk when using a charge-coupled device (CCD) camera. The measured depth of focus of the metalens is 9 μm, obtained from the light intensity distribution along the *z*-axis at *x* = 0 as shown in Fig. S2 in the Supplementary Material. We measured that 31% of the incident laser power reaches the focal plane, representing the transmission of the metalens. The measured focusing efficiency is 93.3%, defined as the ratio of the power within the region with a radius of three times the full width at half maximum around the focus in the focal plane^38^ to the total power reaching the focal plane of the metalens. This high NA can help achieve the necessary deep potential well for stable particle trapping in a vacuum. Details of the trapping potential calculations are provided in the Supplementary Material section S2.

### Nanofabrication of nanorods

**Fig. 2:**
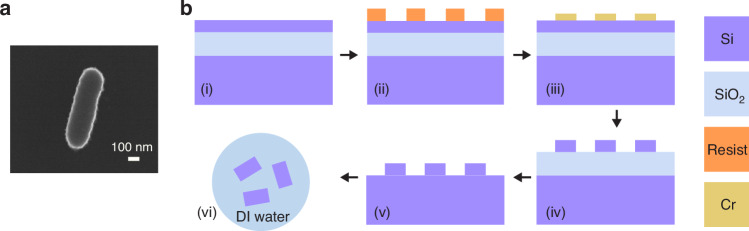
Fabrication flow of nanorods. **a** SEM image of a fabricated nanorod. The nanorod has a width *W*_rod_ of 214 nm, a length *L*_rod_ of 753 nm and a height *H*_rod_ of 220 nm. **b** Nanofabrication process for Si nanorods with a designed shape and size

In this section, we show the nanofabrication of nanorods used in optical levitation in a vacuum. Here, rectangular nanorods are fabricated based on a silicon-on-insulator (SOI) wafer. The process outlined here can be adapted to fabricate particles with any desired shape. Figure 2a shows an SEM image of a fabricated rectangular nanorod. The nanorod has a width *W*_rod_ of 214 nm, a length *L*_rod_ of 753 nm and a height *H*_rod_ of 220 nm.

Figure 2b illustrates the fabrication process of the nanorods. The SOI wafer used in this procedure has a 220 nm thick silicon layer and a 2 μm thick buried oxide layer. The nanorod pattern is transferred to a 300 nm thick ZEP520A resist layer using single-step e-beam lithography (Fig. 2b(ii)). A 40 nm thick Cr layer is deposited by e-beam evaporation, and then the particle patterns are transferred to the Cr surface through a liftoff step (Fig. 2b(iii)). Using Cr as a hard mask, the patterns are then transferred to the Si layer using reactive-ion etching (RIE) (Fig. 2b(iv)). The Cr layer is subsequently removed using a Cr etchant and then the buried oxide layer is removed using HF vapour to release the silicon particles (Fig. 2b(v)). At last, the Si nanorods are transferred from the silicon substrate to deionized (DI) water using an ultrasonic tank (Fig. 2b(vi)). The nanorods dispersed in DI water are suitable for loading with an ultrasonic nebulizer. The fabrication process shown here can also be used for other particle loading methods, such as laser-induced acoustic desorption^39,40^. The HF etching time can be controlled, enabling the fabrication of Si nanoparticles with a well-defined breaking point in SiO_2_.

### Optical levitation experiment

**Fig. 3:**
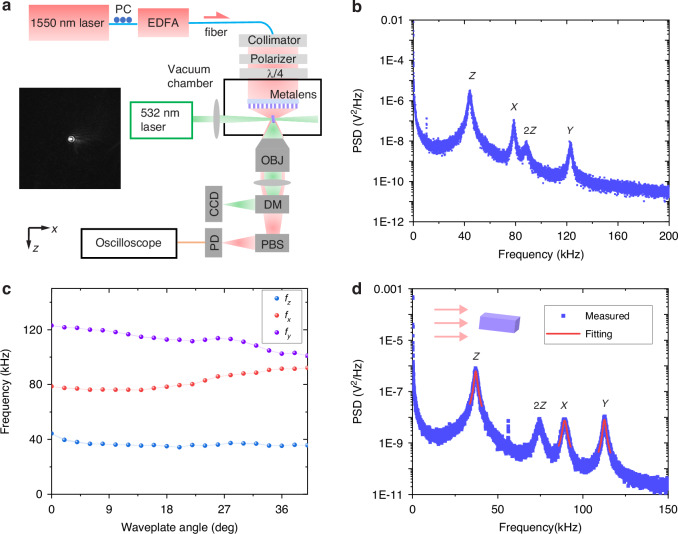
Optical levitation and controlled translation motions. **a** Schematic of the experimental setup for levitating nanofabricated nanorod with metalens. The insert shows the optical image of a levitated nanorod. A 1550 nm laser is focused by a metalens to trap a nanorod inside a vacuum chamber. The light polarization is controlled by a linear polarizer and a quarter-wave plate (QWP). Forward scattered light from the levitated particle, after passing through a polarizing beam splitter (PBS), is sent to a photodetector (PD) for translational and rotational detection. A 532 nm laser is used for imaging the levitated nanorod. **b** Power spectral density (PSD) of the motions of levitated nanorod at pressure *p*_gas_ of 4 mbar. **c** The measured mechanical oscillation frequencies as a function of the laser’s polarization. *f*_*x*_, *f*_*y*_, and *f*_*z*_ are the mechanical oscillation frequencies in the *x*, *y*, and *z* directions, respectively. The QWP angle of 0° means the light is linearly polarized. **d** Lorentzian fittings of measured PSDs of the COM motions for obtaining damping rates of the motions, and the inset shows the orientation of the trapped nanorod

The experimental setup for levitating a nanofabricated nanorod using a metalens is shown in Fig. 3a. A 1550 nm laser, amplified by an erbium-doped fibre amplifier (EDFA), is collimated to a polarizer for obtaining linearly polarized light. A quarter-wave plate (QWP) is utilized to control the light’s polarization state. The metalens, placed inside the vacuum chamber as shown in Fig. 3a, is used to focus the light for trapping. The trapping light scattered by a nanorod is collected by an objective lens and detected by a photodetector to record the nanorod’s motion. A polarizing beam splitter (PBS) is utilized before the photodetector for detecting rotation signals. A focused 532 nm laser is used to illuminate the levitated nanorod for capturing the nanorod’s picture (i.e., the inset picture in Fig. 3a). A dichroic mirror (DM) is used to split the 532 nm and 1550 nm lasers before the detection. Nanorods are loaded into the optical trap in ambient conditions via an ultrasonic nebulizer.

Once a nanorod is trapped, we reduce the vacuum chamber’s pressure *p*_gas_ to 4 mbar. Figure 3b shows the power spectral density (PSD) signal of the particle’s motions when the trapping laser beam is linearly polarized. The PSD is defined as^41^

2 $${S}_{qq}(\omega )=\frac{{\varGamma }_{\rm{CM}}{k}_{B}{T}_{\rm{CM}}/(\pi M)}{{({\omega }^{2}-{\omega }_{q}^{2})}^{2}+{\varGamma }_{\rm{CM}}^{2}{\omega }^{2}}$$

where *Γ*_CM_ is the damping rate of the COM motion, *k*_*B*_ is the Boltzmann constant, *T*_CM_ is the temperature of the COM, *M* is the particle’s mass, and *ω*_*q*_ is the mechanical oscillation frequency of the trapped nanorod. Using Eq. (2) to fit each peak, we can obtain that the oscillation frequencies in the *z*, *x*, and *y* directions are 43.6 kHz, 78.3 kHz, and 122.8 kHz, respectively. The frequency in the *z*-direction is lower than the other frequencies because the optical field is elongated along the direction of the propagating beam. A second harmonic signal along the *z*-direction in the PSD (Fig. 3b) arises from the non-perfectly harmonic trapping potential in the axial direction. The power used for trapping is 30 mW. Using Eqs. (S2) and (S3) in Supplementary Material, the calculated oscillation frequencies along the *x* and *y* directions are 71.3 kHz and 140.7 kHz, respectively. They are close to the measured values, with the error between the calculated and measured values smaller than 12.7%. Using Eq. (S4) in Supplementary Material, the calculated frequency along the *z*-axis is 21.4 kHz, which shows a large difference compared to the measured value (*f*_*z*_ = 43.6 kHz). This discrepancy is because the nanorod’s size along the *z*-axis is 753 nm, and the Rayleigh optics approximation is not valid along this direction. Details of the calculation of translational frequencies are presented in the supplementary material section S3. The levitated subwavelength and high-refractive-index silicon nanorod can be investigated more in the future. For example, Mie resonances in it can introduce additional dynamics control in levitated optomechanics^42^. Compared to silica particles, silicon particles with Mie resonances can offer enhanced performance in terms of trap frequency, trap depth, and optomechanical coupling rates. Moreover, Mie resonances could enable a sign change in the particle’s polarizability by adjusting the laser frequency.

The trapping laser’s focal field depends on the laser beam’s polarization, which can affect the levitated nanorod’s motion. Figure 3c shows that the COM motions of the levitated nanorod can be manipulated by changing the trapping laser beam’s polarization. The waveplate angle in Fig. 3c refers to the angle between the optical axis of the QWP and the polarizing axis of the polarizer. The ellipticity of the polarized light increases with increasing angle. *f*_*x*_, *f*_*y*_, and *f*_*z*_ are the mechanical oscillation frequencies in the *x*, *y*, and *z* directions, respectively. The error bars in Fig. 3c are calculated based on the standard deviation of the measurements. The same method is applied to determine the error bars for the rotation frequencies in the following text. The numerical values of the error bars are provided in Fig. S3 in the Supplementary Material. It can be seen that *f*_*x*_ experiences great increases and *f*_*y*_ experiences great decreases when adjusting the input beam’s polarization from linear to elliptical polarization. When the polarization is changing, the variation in the mechanical oscillation frequency along the *z*-axis is much smaller compared to the changes in frequencies *f*_*x*_ and *f*_*y*_. This is because the field distribution in the *z* direction is not affected by the laser beam’s polarization. The fluctuations in the oscillation frequencies may be due to slight misalignment of the optical setup during the polarization adjustment process.

For an optically levitated nanorod, anisotropic damping rates for COM motions in the air are expected^6,7^. The ratio of damping rates depends on the aspect ratio of the levitated particle. Thus, we use the ratio of damping rates to identify that the levitated particle is a fabricated nanorod. Figure 3d shows the PSD signals of the detected particle motion with a QWP angle of 30° and pressure *p*_gas_ of 4 mbar. The red curves are the Lorentzian fittings based on Eq. (2). A maximum damping ratio *Γ*_*x*_ (14550.3 rad/s)/*Γ*_*z*_ (9917.7 rad/s) of 1.467 is obtained when the QWP angle is 30°. This ratio closely matches the damping ratio calculated for a chain tetramer shown in the supplementary information of ref. ^7^, suggesting a particle with a length-to-width ratio of around 4 is trapped. Since the fabricated nanorod has a length-to-width ratio of 3.52, this indicates that the fabricated nanorod in the chamber is levitated. Also, according to the damping ratio information in the supplementary material of ref. ^7^, the damping rate along the particle’s long axis is the smallest. In our experiments, the measured damping rate along the *z*-axis is the smallest, and the *z*-axis corresponds to the optical axis in our setup. So the nanorod’s long axis is oriented parallel to the optical axis, as shown in the inset of Fig. 3d.

**Fig. 4:**
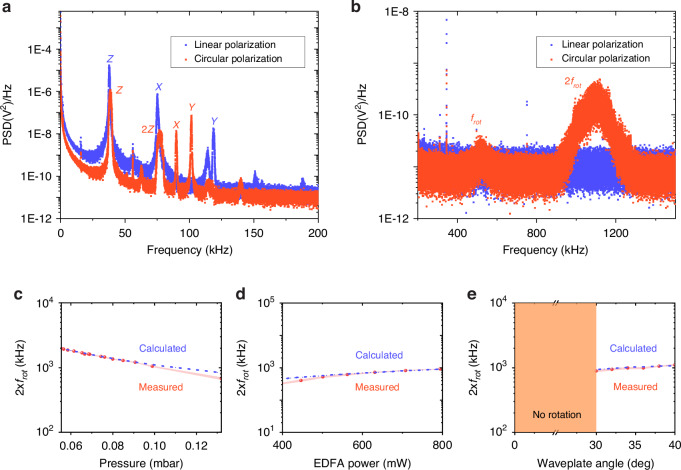
Control of the rotation of a levitated nanorod. **a** Power spectral density (PSD) at pressure *p*_gas_ of 0.11 mbar, with linear (blue line) and circular (orange line) polarization states, respectively. The used polarization angles are 0° and 40°, respectively. The frequency ranges from **a** 0 to 200 kHz and **b** 200–1500 kHz. The rotation frequency’s dependency on air pressure (**c**), laser power (**d**), and polarization (**e**)

In the following, we will demonstrate the control over the rotational dynamics of an optically levitated nanorod by a metalens. The pressure *p*_gas_ inside the vacuum chamber is reduced to 0.11 mbar in order to mitigate the damping effect caused by gas molecules and to observe a clear rotational signal. Figure 4a (4b) shows the PSD spectra from 0 to 200 kHz (200–1500 kHz) for circularly polarized (orange curve) and linearly polarized (blue curve) light beams, respectively. While the translational frequencies along *x*, *y*, and *z* directions in Fig. 4a are similar to that (Fig. 3b) at pressure *p*_gas_ of 4 mbar, the linewidth of the peaks in Fig. 4a is much narrower than that in Fig. 3b. This is because the damping caused by gas molecules decreases with decreasing air pressure^2^.

Figure 4b clearly shows that two new trapping frequencies (*f*_rot_ and 2*f*_rot_) along the *z*-axis appear on the PSD curve for the circularly polarized laser beam, in comparison to the linearly polarized light. As the pressure is reduced further, the frequencies *f*_rot_ and 2*f*_rot_ increase as shown in Fig. 4c. This is a typical feature of the spin rotation of a levitated nanorod. In the PSD spectrum, the amplitude of 2*f*_rot_ is much larger than that of *f*_rot_. The large amplitude of 2*f*_rot_ is due to the geometrical symmetry of the nanofabricated nanorod^7,36^. The small *f*_rot_ is attributed to the small deviation of the perfect geometry symmetry.

The rotation of the levitated nanorod can be attributed to the torque exerted by the circularly polarized light. The strength of this optical torque *τ*_*z*_ depends on the laser power and the particle’s polarizability, and can be expressed as^43^

3 $${\tau }_{z}=\frac{1}{2}{E}_{0}^{2}\frac{{k}^{3}}{6\pi {\varepsilon }_{0}}{(\Delta {\alpha }_{0})}^{2}$$

where *E*_*0*_ is the amplitude of the optical input field, *k* is the wavenumber, *ε*_*0*_ is the dielectric constant in vacuum, and Δ*α*_*0*_ is given by

4 $$\Delta {\alpha }_{0}=\frac{{\alpha }_{x}}{1+i{k}^{3}{\alpha }_{x}/(6\pi {\varepsilon }_{0})}-\frac{{\alpha }_{y}}{1+i{k}^{3}{\alpha }_{y}/(6\pi {\varepsilon }_{0})}$$

with *α*_*x*_ and *α*_*y*_ being the nanorod’s polarizability along the *x*- and *y*-axis, respectively. The maximum steady-state rotation frequency of the particle can be represented as

5 $${f}_{rot}=\frac{{\tau }_{z}}{2\pi I\varGamma }$$

where *I* is the rectangular nanorod’s momentum of inertia, and *Γ* is the rotational damping rate for diffuse reflection of gas molecules. The equations for the nanorod’s damping rate and momentum of inertia are presented in Eq. (S6) and Eq. (S7) in the Supplementary Material, respectively.

Figure 4c shows the calculated rotation frequency (blue curve) of the nanorod at different pressures using Eqs. (3–5). More details of the calculation are shown in the Supplementary Materials section S4. For complex-shaped nanoparticles, their optical torque *τ*_*z*_ can be calculated by combining the finite difference in the time-domain method with the discrete dipole approximation method^44^. The calculated rotation frequency closely matches the experimentally measured frequency, when using the nanorod width *W*_rod_ of 216.2 nm which is 2.2 nm different from the measured nanorod width of 214 nm. All the parameters used for calculation are listed in Table S1 in the Supplementary Material.

The dependency of the nanorod’s rotation frequency on the laser beam’s power and polarization is experimentally explored as shown in Fig. 4d, e. Figure 4d shows that the measured rotational frequency is proportional to the laser power, which is consistent with Eq. (3). The polarization is tuned by rotating the QWP. As shown in Fig. 4e, when the angle is within the range from 0 to 30°, there is no rotational signal in the PSD spectrum, indicating that the optical torque applied to the nanorod is smaller than the air drag. When the QWP angle is larger than 30°, the nanorod starts to rotate and the rotational PSD signal appears. With furtherly increasing the QWP angle, more spin angular momentum can be transferred to the nanorod, thereby a higher rotation frequency can be obtained. The blue curves in Fig. 4d, e show the calculated results using the parameters listed in Table S1 in Supplementary Material, which agree well with the experimental measurements (orange curves).

## Discussion and conclusion

Here, we demonstrated the optical levitation of a nanofabricated nanorod in a vacuum environment using nanofabricated high-NA metalens. Controllable COM motions and rotation of the levitated nanorod have also been demonstrated. The levitated nanorod can achieve a rotation frequency of around 1 MHz.

In this work, we mainly focused on the demonstration of the feasibility of combining nanofabricated metalens and nanofabricated nanoparticles for optical levitation in a vacuum. The achieved MHz rotation, similar to the rotation frequency of a nanorod levitated by two conventional lenses^36^, has not reached the state-of-the-art GHz rotation^6,7^. One reason is the small polarizability due to the small size difference in the nanorod’s width and height. Increasing the difference in the nanorod’s width and height could increase the rotational frequency. Achieving state-of-the-art rotation also requires stable trapping of the particle in a high vacuum. High focusing efficiency of the metalens and feedback control of the particle can be implemented in the future. In the experiments, the trapped nanorod would be lost easily when the waveplate angle is 45°. The loss mechanism may be related to the particle’s shape, size and its motion under circularly polarized input light. Future investigations could also include studying the motion of a levitated particle when circularly polarized light is used, the impact of polarization on the orientation of a levitated particle by the metalens, as well as the effects of particle shapes (such as rods and dumbbells) and sizes on levitation.

In our experiment, a metalens is utilized to achieve a single-point focus of the light beam. In future studies, the metalens can combine single-point focusing with an adjustable focus^45^ to explore short-range forces or with vortex light fields^23,46^ to stably trap large-sized particles for enhancing acceleration detection sensitivity, in a high vacuum. In addition, single focal points can be extended to multi-focal points^34^ for research into macroscopic many-body quantum mechanics.

The nanofabrication techniques are not limited to the fabrication of the nanorods. The proposed method, combining methods^47^ for reliable fabrication of nanostructures with sub-10 nm features in the future, can be utilized to fabricate nanoparticles of any shape and size, enabling the highlight of specific motions of a levitated particle and the exploration of novel particle manipulation techniques. For example, anisotropic nanoparticles together with versatile control of libration motions can be explored for nanoscale gyroscopes and studying macroscopic rotational quantum physics^48–50^. Triangular or hexagonal prisms can be used to achieve polarization-based inverse optical torque^25^, or for exploring high-frequency gravitational wave detection^24^. In this proof-of-concept experiment, we employed e-beam lithography to define the size and shape of nanorods. For high-throughput and low-cost fabrication of particles, conventional photolithography can be used^51^.

The demonstrated translation and rotation of nanofabricated particles in a vacuum based on metalens can combine the powerful light field control capability of the metalens with the customization advantage of nanoparticles. This can provide an ideal platform for further expanding the applications of optical levitation. For example, multi-foci metalens can simultaneously levitate particles of different shapes, enabling on-chip sensing of multiple parameters such as force, acceleration, and torque. Additionally, it can be used to study coupling effects between different levitated particles to explore collective quantum phenomena^19^ and particle assembly^52^. Meanwhile, this system using nanofabricated ultrathin metalens can provide a compact solution for integrated on-chip sensing applications, such as acceleration (translation) and torque (rotation). In the future, this can be combined with chip-based light source^53^ and vacuum packaging technology^54^ to realize a miniaturized, robust, and scalable on-chip integrated optical levitation system. This approach holds the potential to significantly transit vacuum optical levitation systems from the laboratory into practical applications.

## Materials and methods

The designed metalens is fabricated using a nanofabrication process. Firstly, an 800 nm thick a-Si layer is deposited on a glass substrate using a plasma-enhanced chemical vapour deposition tool. Then, the metalens is patterned using electron beam (e-beam) lithography, followed by reactive-ion etching of 800 nm into the a-Si layer. Figure 1d presents a top-view scanning electron microscopy image of the central region of the fabricated metalens.

## Supplementary information

Supplementary material

## Data Availability

The data from this paper can be obtained from the University of Southampton ePrints research repository (10.5258/SOTON/D3356).
