# Supplementary material for: Levitation and controlled MHz rotation of a nanofabricated rod by a high-NA metalens
Source: Microsyst Nanoeng. 2025 Apr 21;11:67. doi: 10.1038/s41378-025-00886-7 (PMC12012181; doi:10.1038/s41378-025-00886-7)
Supplement: Supplementary file 1 — Supplementary material [file 41378_2025_886_MOESM1_ESM.docx]

**Levitation and controlled MHz rotation of a nanofabricated rod by a high-NA metalens**

Hailong Pi,^1^ Chuang Sun,^1^ Kian Shen Kiang,^1^ Tiberius Georgescu,^2^ Bruce (Jun-Yu) Ou,^2^ Hendrik Ulbricht,^2^ Jize Yan^1^*

^1^School of Electronics and Computer Science, University of Southampton, SO17 1BJ, UK.

^2^School of Physics and Astronomy, University of Southampton, Southampton, SO17 1BJ, United Kingdom.

*Corresponding Authors: J.Yan@soton.ac.uk

**S1. Simulated propagation phase and transmission for nanopillars using different polarizations**

Figure S1 shows the simulated propagation phase and transmission of the nanopillar as functions of the nanopillar's side length *W* under various input polarization states, including linear polarization along the *x*-axis, linear polarization along the diagonal of the nanopillar’s square cross-section and circular polarization. The figure shows that the simulated transmission and phase response for the nanopillars are the same under different polarizations.


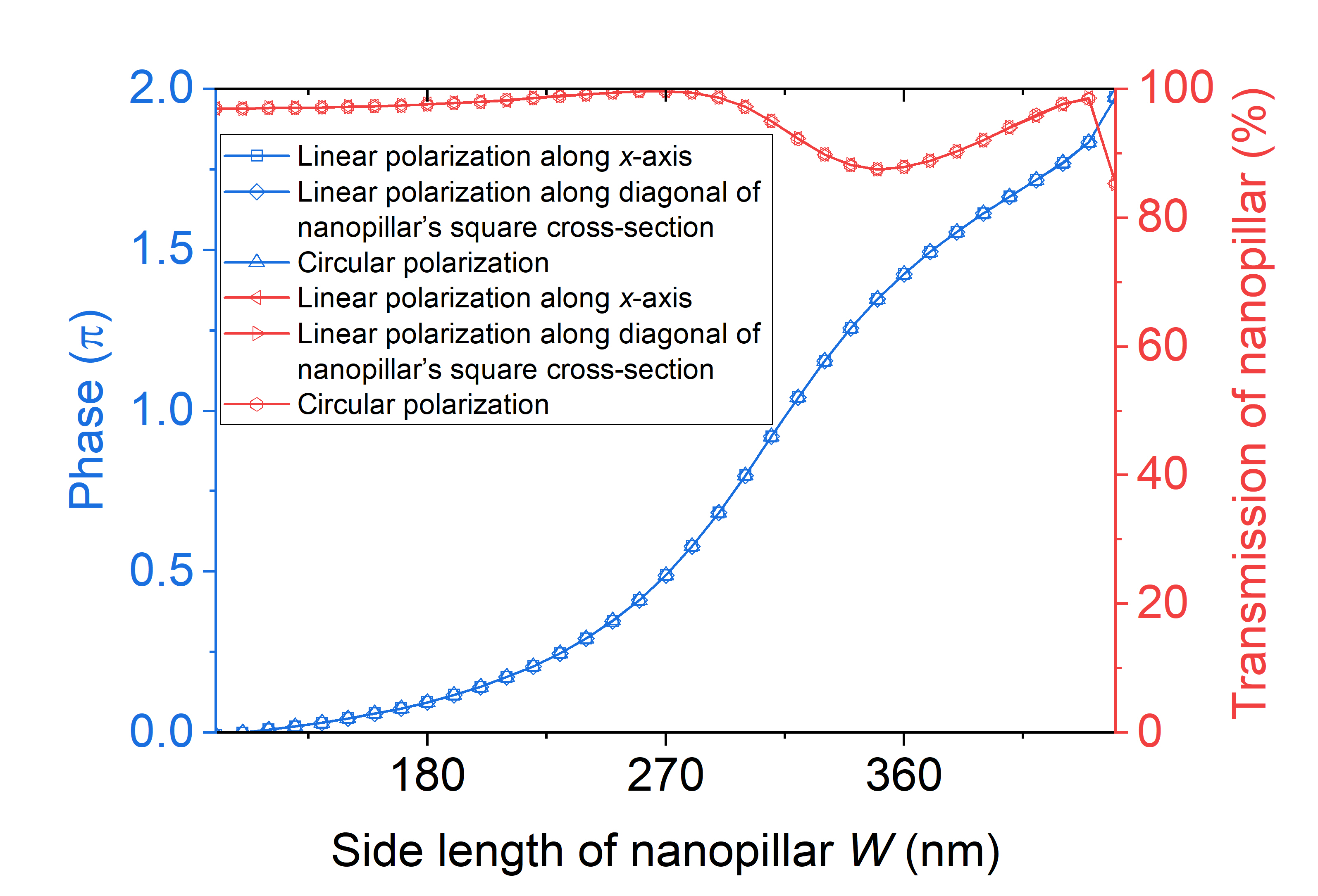


Fig. S1 Simulated propagation phase and transmission as functions of the nanopillar's side length *W* under different input polarizations.

**S2. Trapping potential at the focus**

In optical levitation, achieving stable trapping requires that the trapping potential is at least 10 times greater than the average kinetic energy of the trapped sphere^1^. The average kinetic energy is given by *k_B_T*, and *k_B_* is Boltzmann constant and *T* is the temperature of the surrounding medium. For a silicon sphere particle with a radius *R*, the trapping potential can be expressed as^2^

|  | $U=-\frac{2R^{3}}{c}(\frac{n_{r}^{2}-1}{n_{r}^{2}+2})\frac{2P}{w_{0}^{2}}$ | (S1) |
| --- | --- | --- |

where *c* is the propagation speed of light in a vacuum, *n_r_* is the effective index of the particle, *P* is the laser power at the focus point, and *w_0_* is the beam waist radius which is inversely proportional to the numerical aperture (NA). Therefore, with a larger NA, the beam waist radius *w_0_* at the focus becomes smaller, resulting in a deeper trapping potential. This makes it easier to achieve the condition where the trapping potential is greater than 10*k_B_T.*

**S3. Calculation of the nanorod’s translational frequencies**

The equations for calculating translational frequencies in the *x*, *y* and *z* directions can be expressed as^2^

|  | $\omega_{x}^{2}=\frac{4\alpha_{x}P}{\pi\varepsilon_{0}cw_{x}^{4}M}$ | (S2) |
| --- | --- | --- |
|  | $\omega_{y}^{2}=\frac{4\alpha_{y}P}{\pi\varepsilon_{0}cw_{y}^{4}M}$ | (S3) |
|  | $\omega_{z}^{2}=\frac{2\alpha_{z}P}{\pi\varepsilon_{0}cw_{0}^{2}z_{0}^{2}M}$ | (S4) |

where *α_j_* is the polarizability of the particle along the *j* axis. *P* is the power of the input light. *ε_0_* is the dielectric constant in a vacuum, *c* is the propagation speed of light in a vacuum, *w_j_* is the waist radius along the *j*-axis at the focus point, *z_0_* is the Rayleigh range, *λ_0_* is the laser wavelength and *M* is the mass of the particle.

The polarizability of the particle along the *j* axis used in Eq. (S2-S4) can be expressed as^2,3^

|  | $\alpha_{j}=\frac{\varepsilon_{0}V}{L_{j}+\frac{1}{n^{2}-1}}$ | (S5) |
| --- | --- | --- |

where

$$L_{x}=\int_{0}^{\infty} \frac{abcds}{2(s+a^{2})^{3/2}(s+b^{2})^{1/2}(s+c^{2})^{1/2}}$$

$$L_{y}=\int_{0}^{\infty} \frac{abcds}{2(s+a^{2})^{1/2}(s+b^{2})^{3/2}(s+c^{2})^{1/2}}$$

$$L_{z}=\int_{0}^{\infty} \frac{abcds}{2(s+a^{2})^{1/2}(s+b^{2})^{1/2}(s+c^{2})^{3/2}}$$

where *a*, *b* and *c* are the half values of the nanorod’s width *W_rod_*, height *H_rod_* and length *L_rod_*.

A laser beam with linear polarization focused by a high-NA lens will have an asymmetric intensity distribution, with an elongated field distribution along the polarization than along the other transverse directions^4^. The asymmetric intensity distribution in the *x*-*y* plane will cause the levitated particle to have non-degenerate translational eigenfrequencies in the *x* and *y* directions. The intensity distribution of the focused linear polarized plane wave can be calculated using the Richards-Wolf formulas^4^. The calculated *w_x_* is 875 nm and *w_y_* is 619 nm, when the 1550 nm light polarized along *x* direction is focused by a lens with NA = 0.953.


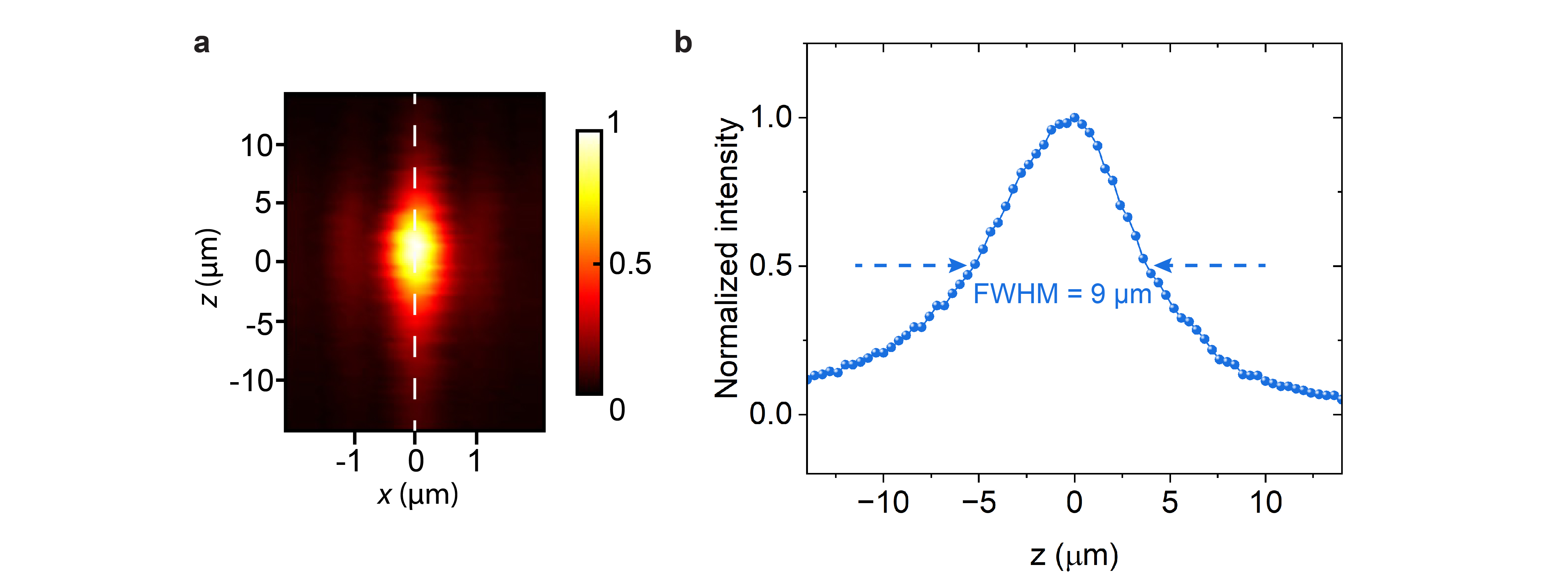


Fig. S2 (a) Measured laser intensity distribution along the *z*-direction. (b) Light intensity distribution along the *z*-axis at *x*=0 (dotted line in (a)).

We estimated the power at the focus point to be 30 mW, considering the optical losses from components such as fiber connectors, polarizer, flange viewport and metalens in the experimental setup. Using this power to the above Eq. (S2) and (S3), the calculated oscillation frequencies in the *x* and *y* directions are 71.3 kHz, and 140.7 kHz, respectively. They are close to the measured frequencies, with the error between the calculated and measured values smaller than 12.7 %. Using Eq. (S4), the calculated frequency along the *z*-axis is 21.4 kHz, by using the Rayleigh range as half of the measured depth of focus (9 μm) of the metalens. The depth of focus is obtained from the measured light intensity distribution along the *z*-axis at *x*=0 as shown in Fig. S2. The calculated oscillation frequency shows a difference compared to the measured value (*f_z_*=43.6 kHz). This discrepancy is because the nanorod's size along the *z*-axis is 753 nm, and the Rayleigh optics approximation is not valid along this direction.

**S4. Calculation of nanorod’s rotation frequency**

The rotation of levitated nanorods is induced by the torque transferred from the circularly polarized light. Equation (3) in the main text shows that under circularly polarized input, the optical torque *τ_z_* depends on the intensity of the input light *P* and the polarizability of the particle *Δα_0_*. The maximax steady-state rotation frequency of the particle depends on the optical torque *τ_z_*, rotation-damping rate *Γ* and nanorod’s momentum of inertia *I*, as shown in Eq. (5) in the main text.

The rotation-damping rate *Γ* for the diffuse reflection of gas molecules is^5^

|  | $\Gamma=\frac{HLp_{gas}}{2M}\sqrt{\frac{2\pi m_{g}}{k_{B}T}}\left(\frac{3}{2}+\frac{\pi}{4}\right)$ | (S6) |
| --- | --- | --- |

The rectangular nanorod’s momentum of inertia *I* is

|  | $I=\frac{1}{12}M\left(W_{rod}^{2}+H_{rod}^{2}\right)$ | (S7) |
| --- | --- | --- |

where the *W_rod_*, *H_rod_* and *L_rod_* are the nanorod’s width, height and length respectively. *p_gas_* is the pressure inside the vacuum chamber, *M* is the mass of the nanorod, *m_g_* is the mass of the gas molecule, *k_B_* is the Boltzmann constant, *T* is the gas temperature. All the parameters used in the calculation for Fig. 4(c-e) are listed in Table S1 below.s

Table S1: List of all the calculation parameters

| **Parameters** | **Quantity** | **Value** | **Unit** |
| --- | --- | --- | --- |
| Nanorod width | *W_rod_* | 216.2 | *nm* |
| Nanorod height | *H_rod_* | 220 | *nm* |
| Nanorod length | *L_rod_* | 753 | *µm* |
| Dielectric constant in a vacuum | *ε_0_* | 8.85×10^-12^ | *F/m* |
| Trapping power | *P* | 0.03 | *W* |
| Beam waist radius | *w_0_* | 692 | *nm* |
| Light speed in a vacuum | *c* | 3×10^8^ | *m/s* |
| Refractive index of silicon | *n_r_* | 3.476 |  |
| Laser wavelength | *λ_0_* | 1550 | *nm* |
| Density of silicon | *ρ* | 2330 | *kg/m^3^* |
| mass of the gas molecule | *m_g_* | 4.68×10^-26^ | *kg* |
| Boltzmann constant | *k_B_* | 1.38×10^-23^ | *J/K* |
| Temperature | *T* | 300 | *K* |
| Pressures in Fig. 4d/4e experiments | *p_gas_* | 0.12 / 0.10 | *mbar* |

**S5. Values of error bars in the main text**

The figure below shows the values of the error bars used in Fig. 3(c) and Fig. 4(c-e).


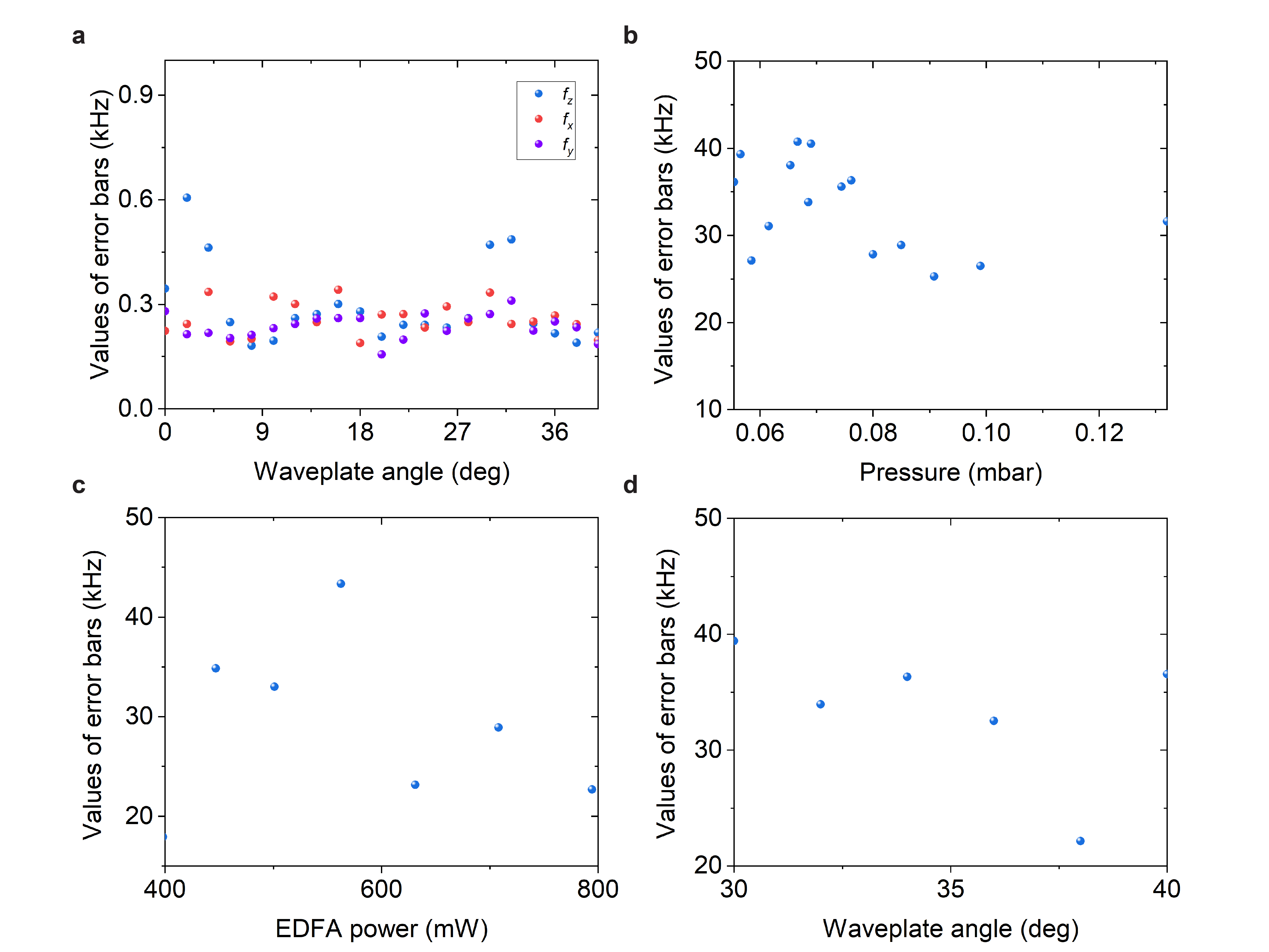


Fig. S3 The values of error bars used in Fig. 3c (a), Fig. 4c (b), Fig. 4d (c) and Fig. 4e (d).

**References**

1 Ashkin, A., Dziedzic, J. M., Bjorkholm, J. E. & Chu, S. Observation of a single-beam gradient force optical trap for dielectric particles. *Opt. Lett.* **11**, 288-290, (1986).

2 Ahn, J. *Spin Optomechanics of Levitated Nanoparticles*, Purdue University Graduate School, (2020).

3 Bohren, C. F. & Huffman, D. R. *Absorption and scattering of light by small particles*. (John Wiley & Sons, 2008).

4 Richards, B., Wolf, E. & Gabor, D. Electromagnetic diffraction in optical systems, II. Structure of the image field in an aplanatic system. *Proceedings of the Royal Society of London. Series A. Mathematical and Physical Sciences* **253**, 358-379, (1959).

5 Kuhn, S. *et al.* Full rotational control of levitated silicon nanorods. *Optica* **4**, 356-360, (2017).
